# Physical activity of moderate-intensity optimizes myocardial citrate cycle in a murine model of heart failure

**DOI:** 10.3389/fphys.2025.1568060

**Published:** 2025-04-02

**Authors:** Lucyna Widacha, Joanna Szramel, Zenon Nieckarz, Anna Kurpinska, Ryszard T. Smolenski, Stefan Chlopicki, Jerzy A. Zoladz, Joanna Majerczak

**Affiliations:** ^1^ Chair of Exercise Physiology and Muscle Bioenergetics, Faculty of Health Sciences, Jagiellonian University Medical College, Krakow, Poland; ^2^ Department of Experimental Computer Physics, Marian Smoluchowski Institute of Physics, Faculty of Physics, Astronomy and Applied Computer Science, Jagiellonian University, Krakow, Poland; ^3^ Jagiellonian Centre of Experimental Therapeutics (JCET), Jagiellonian University, Krakow, Poland; ^4^ Department of Biochemistry, Faculty of Medicine, Medical University of Gdansk, Gdansk, Poland; ^5^ Chair of Pharmacology, Faculty of Medicine, Jagiellonian University Medical College, Krakow, Poland

**Keywords:** exercise tolerance, oxidative metabolism, citrate, branched-chain amino acids, tricarboxylic acid cycle intermediates

## Abstract

**Introduction:**

There is growing body of evidence that an enhanced concentration of branched-chain amino acids (BCAAs), as a consequence of an impaired myocardial oxidative metabolism, is involved in the occurrence and progression of heart failure (HF). The purpose of this study was to examine the effect of 8 weeks of spontaneous wheel running (8-sWR) (reflecting low-to-moderate intensity physical activity) on the myocardial [BCAAs] and mitochondrial oxidative metabolism markers, such as tricarboxylic acid (TCA) cycle intermediates (TCAi), mitochondrial electron transport chain (ETC) proteins and mitochondrial DNA copy number (mtDNA/nDNA) in a murine model of HF.

**Methods:**

Adult heart failure (Tgα_q_*44) and wild-type (WT) mice were randomly assigned to either the sedentary or exercising group. Myocardial concentrations of [TCAi] and [BCAAs] were measured by LC-MS/MS, ETC proteins were determined by Western immunoblotting and mtDNA/nDNA was assessed by qPCR.

**Results:**

Heart failure mice exhibited decreased exercise performance capacity as reflected by a lower total distance covered and time of running in wheels. This was accompanied by impaired TCA cycle, including higher citrate concentration and greater [BCAAs] in the heart of Tgα_q_*44 mice compared to their control counterparts. No impact of disease at its current stage i.e., in the transition phase from the compensated to decompensated stage of HF on the myocardial mitochondrial ETC, proteins content was observed, however the altered basal level of mitochondrial biogenesis (lower mtDNA/nDNA) in the heart of Tgα_q_*44 mice compared to their control counterparts was detected. Interestingly, 8-sWR significantly decreased myocardial citrate content in the presence of unchanged myocardial [BCAAs], ETC proteins content and mtDNA copy number.

**Conclusion:**

Moderate-intensity physical activity, even of short duration, could be considered an effective intervention in heart failure. Our results suggest that central metabolic pathway - TCA cycle appears to be more sensitive to moderate-intensity physical activity (as reflected by the lowering of myocardial citrate concentration) than the mechanism(s) regulating the BCAAs turnover in the heart. This observation may have a particular importance in heart failure, since an improvement of impaired myocardial oxidative metabolism may contribute to the upgrading of the clinical status of patients.

## 1 Introduction

Heart failure (HF), which develops predominantly as a consequence of coronary artery disease and chronic hypertension, is currently recognized as a global pandemic (Ambrosy et al., 2014). The most prominent functional symptom of heart failure is exercise intolerance, which is caused mainly by an impairment of the heart as a pump, resulting in decreased cardiac output and limited oxygen supply to tissues, in turn leading to energy deficiency in the locomotory muscles (Karwi et al., 2019; Takada et al., 2022). Regardless of the main type of heart failure (HF with preserved ejection fraction vs. HF with reduced ejection fraction), the ability to increase cardiac output with exertion is frequently abnormal (Omote et al., 2022). Therefore, the level of exercise tolerance, especially its improvement after treatment and/or physical training, is one of the hallmarks of upgrading heart function and clinical status of the patient (Nijholt et al., 2022). Currently, a major clinical challenge in heart failure is to develop strategies to preserve or enhance the pump function of the heart while maintaining cell viability (Ingwall, 2002).

The pathophysiology of heart failure is still not entirely understood (Piepoli et al., 2001), however, mitochondrial dysfunction in the failing heart, reflected by an impairment in the rate of ATP resynthesis in the oxidative phosphorylation process (OXPHOS) and accompanied by mitochondrial structure remodeling (Rosca et al., 2013), is now recognized as a key alteration in this disease (Lopaschuk et al., 2021). Mitochondrial dysfunction in heart failure seems to be a result of maladaptation to energy stress in the heart, which in consequence leads to cardiac damage (Zhou and Tian, 2018).

In the normal heart at rest, mitochondrial OXPHOS (along with tricarboxylic acid cycle) is responsible for approximately 95% of ATP production, whereas anaerobic glycolysis accounts for only ∼5% of ATP resynthesis (Ingwall, 2002; Duncker et al., 2019; Lopaschuk et al., 2021). Therefore, a decrease in oxygen supply to myocardium may lead to cardiac failure due to an impairment of ATP resynthesis.

Under physiological conditions, fatty acids (FFAs) are the main oxidative substrate for ATP resynthesis in the heart (∼85%) (Murashige et al., 2020; Lopaschuk et al., 2021). In the failing heart, FFA oxidation is significantly reduced (by ∼40% (Osorio et al., 2002)), and myocardial fuel shifts from FFAs to oxidation of ketones (from ∼10 to 15% to ∼20–30% in healthy and failing hearts, respectively), glucose (from ∼2 to 8% to ∼5–10% in healthy and failing hearts, respectively), lactate and amino acids (Aubert et al., 2016; Murashige et al., 2020; Lopaschuk et al., 2021). Furthermore, the myocardial ATP resynthesis in heart failure at rest is shifted toward greater involvement of anaerobic glycolysis, *i.e.*, from ∼5 to 7% in healthy hearts to ∼13–19% in hypertrophied hearts (Allard et al., 1994). Furthermore, the phosphocreatine (PCr) pool (direct source of ATP resynthesis) in the failing heart is decreased and contributes to reduced cardiac function (Liao et al., 1996; Zhou and Tian, 2018).

As recently noted, branched-chain amino acids (BCAAs), despite being rather small contributors to ATP resynthesis in the heart (∼1–2% in the healthy heart and <1% in the failing heart (Lopaschuk et al., 2021)), play a significant signaling role in the pathophysiology of heart failure. BCAAs (leucine, valine, isoleucine) are essential amino acids for mammals (i.e., must be obtained from dietary proteins). BCAAs are either used to synthesize new proteins or imported to the mitochondria (mainly in the striated muscles and liver) where they are oxidized to fuel the tricarboxylic acid (TCA) cycle (Neinast et al., 2019). The accumulation of BCAAs (especially when their oxidation is compromised) is currently recognized as one of the hallmarks of heart failure (Li et al., 2019; Uddin et al., 2019), since as discovered, elevated myocardial BCAA level (as a consequence of impaired oxidative metabolism) trigger mammalian target of rapamycin (mTOR) protein kinase activation, leading to adverse heart remodeling and a decrease in myocardial contractility (Shao et al., 2018; Karwi et al., 2019; Karwi and Lopaschuk, 2023). On the other hand, while myocardial and circulating BCAA accumulation is associated with the progression of heart failure (Li et al., 2019), intensified BCAA oxidation, exerts a cardioprotective role in heart failure (Murashige et al., 2022).

Interestingly, it has been demonstrated in human studies that prolonged exercise (Blomstrand et al., 1988) or even short-term (a few minutes) but high-intensity exercise (∼70% of maximal oxygen uptake) (Gawedzka et al., 2020) decreases the circulating BCAA concentration. Therefore physical activity, as non-pharmacological treatment, seems to have a beneficial impact on the BCAA oxidation in the heart, through their oxidation in TCA cycle. However, data regarding the effect of physical training on the circulating BCAA levels are inconsistent. It has been demonstrated that physical training exerted no effect on systemic BCAAs concentration, both in humans (Gawedzka et al., 2020) and in animals (Yoshida et al., 2017; Sahu et al., 2024). Furthermore, it has also been demonstrated that a swimming training in mice lasting 8 weeks increased (by ∼20%) circulating levels of BCCAs (Wu et al., 2022).

In the present study, we aimed to show the impact of heart failure on the myocardial BCAAs concentration in relation to oxidative metabolism markers including the TCA cycle intermediates content ([TCAi]) (including citrate) and oxidative phosphorylation (OXPHOS) markers such as mitochondrial electron transport chain (ETC) proteins content and mitochondrial DNA copy number (mtDNA-to-nDNA ratio). The main aim of the present study was to evaluate the impact of 8 weeks of spontaneous wheel running (8-sWR) on the myocardial [BCAAs] in a murine model of heart failure (Tgα_q_*44) in relation to afore-mentioned myocardial oxidative metabolism markers. Accordingly, we aimed to verify whether spontaneous wheel running, which reflects moderate-intensity physical activity (as frequently recommended for heart failure patients), can exert cardioprotective effects in heart failure through an improvement of myocardial oxidative metabolism leading to a decrease in myocardial BCAAs levels.

## 2 Materials and methods

### 2.1 Animals

A total of 77 adult (10-months old at the start of the study, 12-month old at the end of the experiment) female homozygous transgenic Tgα_q_*44 (Tg mice) and age-matched FVB wild-type mice (WT mice) were randomly assigned to either the sedentary (Sed) or trained (Tre) group. Three FVB and 2 Tgα_q_*44 mice died during the experiment and were excluded from the initial groups. Therefore, the final size of the groups were as follows: WT-Sed, n = 21; WT-Tre, n = 16; Tg-Sed, n = 21; and Tg-Tre, n = 14, giving a total number of 72 individuals.

The transgenic Tgα_q_*44 mice (maintained in the FVB background) are characterized by a slowly developing chronic heart failure resulting from the cardiac-specific overexpression of a constitutively active G_q_α* protein that imitates excessive neurohormonal stimulation of the heart and in turn leads to dilated cardiomyopathy (Mende et al., 2001; Grassi et al., 2017; Bardi et al., 2019; Tyrankiewicz et al., 2021a; Tyrankiewicz et al., 2021b). As presented in our previous study, 10-month old Tgα_q_*44 mice have significantly lower stroke volume (by ∼38%) and ejection fraction (by ∼38%) compared to the age-matched control (WT) mice (Grassi et al., 2017). Tgα_q_*44 mice, that mirror the chronic human HF (Bardi et al., 2019; Tyrankiewicz et al., 2021a; Tyrankiewicz et al., 2021b), exhibit the characteristics of delayed progression of heart failure, *i.e.,* even though cardiac systolic and diastolic functions begin to deteriorate at 4–6 months of age, cardiac decompensation usually occurs at ∼12–14 months of age (Mende et al., 2001; Bardi et al., 2019). This progressive impairment in left ventricular function of Tgα_q_*44 mice was previously described by others (Bardi et al., 2019; Tyrankiewicz et al., 2021a; 2021b). In Table 1, we summarize the cardiac function (left ventricular) of Tgα_q_*44 mice at different ages, including 12-month old mice, based on the data presented by us in Bardi et al. (Bardi et al., 2019). As recently demonstrated 12-month old Tgα_q_*44 mice are also characterized by impaired right ventricular function accompanied by alteration in hepatic blood flow and liver congestion (Wojnar-Lason et al., 2024). Moreover, in Table 1 we also present the comparison of sedentary Tgα_q_*44 mice with trained Tgα_q_*44 mice at given age.

**TABLE 1:** Characteristics of cardiac performance (left ventricle) in a murine model of heart failure (Tgα_q_*44 mice) at 6, 12 and 14 months of age in the sedentary and trained mice (8 weeks of spontaneous wheel running) (based on Bardi et al. (2019).

Tgα_q_*44 mice						
	6 months		12 months^a^		14 months	
	Sedentary	Trained	Sedentary	Trained	Sedentary	Trained
**SV (μL)**	29.2 ± 0.7	35.2 ± 0.7^#^	27.4 ± 2.4	33.7 ± 1.1^#^	32.6 ± 2.0	32.5 ± 1.1
**EF (%)**	59.5 ± 1.4	58.7 ± 1.4	46.5 ± 4.4 *	53.0 ± 1.1^#^	51.5 ± 2.9	53.6 ± 3.2
**EDV (μL)**	49.2 ± 1.1	60.3 ± 1.6^#^	59.3 ± 3.4	63.7 ± 2.0	65.1 ± 4.7 *	62.6 ± 4.4
**ESV (**μ**L)**	20.0 ± 0.9	25.0 ± 1.3^#^	31.9 ± 3.2	29.9 ± 1.3	32.5 ± 3.5 *	30.1 ± 3.8
**ER (SV/RR)**	2.91 ± 0.13	3.15 ± 0.10^#^	3.65 ± 0.38	4.03 ± 0.24^#^	4.02 ± 0.18 *	3.99 ± 0.10
**IVCT (% RR)**	12.9 ± 0.6	15.2 ± 0.7	32.7 ± 5.0 *	33.5 ± 2.4	36.1 ± 2.9 *	37.6 ± 2.7
**HR (bpm)**	491 ± 9	499 ± 3	348 ± 14 *	361 ± 9	321 ± 7 *	321 ± 6

*The symbol represents the significant difference between Tgα_q_*44 mice at given age as compared to 6 months old Tgα_q_*44 mice (*P* < 0.05).

^#^The symbol represents the significant difference between trained and sedentary Tgα_q_*44 mice at given age (*P* < 0.05); The symbol.

^&^represents the data obtained for the mice of the same age as in the present study (10 months old at the start of the experiment). *Abbreviations:* SV, stroke volume; EF, ejection fraction; EDV, end diastolic volume; ESV, end systolic volume; ER, Ejection Rate (ER, was normalized to individual SV, and R-R values (R-R peak intervals)), IVCT, isovolumic contraction time; HR, heart rate.

Mice were bred in the Animal Laboratory of the Medical Research Centre of the Polish Academy of Sciences (Warsaw, Poland). Like in our previous work, due to the long-term development of the end-stage phenotype of HF, only female mice were used in the present study, as male mice were too aggressive to keep them in groups for over 10 months (Grassi et al., 2017; Bardi et al., 2019; Tyrankiewicz et al., 2021a). The body weights of the mice were as follows: WT-Sed 32.0 ± 7.0 g, Tg-Sed 32.0 ± 7.3 g, WT-Tre 31.7 ± 4.2 g, and Tg-Tre 29.8 ± 4.1 g.

Throughout the study, the mice were kept one per cage (floor area of 355 × 235 × 190 mm) in a room with a 12 h/12 h light/dark cycle and controlled temperature (22°C–24°C) with *ad libitum* access to water and rodent chow (Altromin 1320; composed of 11% fat, 24% protein, 65% carbohydrates). The cages of trained mice were equipped with running wheels and an electronic system (Columbus Instruments, Columbus, OH, United States), allowing for registration of spontaneous physical activity of mice (all running episodes were recorded at 10 s intervals).

Experimental blinding and randomization strategies were applied when possible. The principal investigator was the only person aware of the group allocation. A sample size between 14 and 21 in each group was evaluated for each particular measurement on the basis of the results obtained from our previous studies.

**FIGURE 1:**
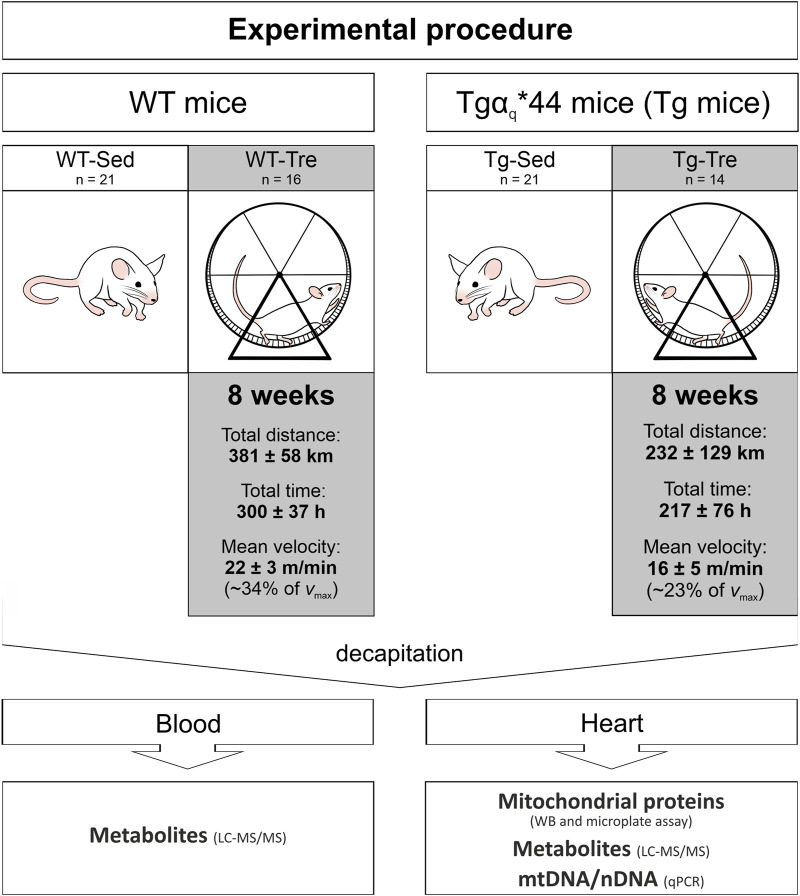
The description of the experimental procedure. *Abbreviations:* WT-Sed and WT-Tre, wild-type (WT) sedentary and trained group of mice, respectively; Tg-Sed and Tg-Tre, Tgα_q_*44 sedentary and Tgα_q_*44 trained group of mice; LC-MS/MS, liquid chromatography coupled with tandem mass spectrometry; WB, western immunoblotting; mtDNA/nDNA, mitochondrial DNA to nuclear DNA ratio; *v*_max_, maximal running velocity.

Animals from the trained groups (WT-Tre and Tg-Tre) were subjected to 8 weeks of spontaneous wheel running (8-sWR), whereas mice from the sedentary groups (WT-Sed and Tg-Sed) did not have access to the running wheels (Figure 1).

Eight weeks after starting the training, all the mice (Sed and Tre) were decapitated after cervical dislocation. All efforts were made to minimize suffering.

The experimental protocols were conducted according to the Guidelines for Animal Care and Treatment of the European Union (EU Directive 2010/63/EU for animal experiments) and were approved by the second Local Institutional Animal Care and Use Committee in Krakow (Permit Number: 197/2018 and 37/2013). All methods in this study were reported in accordance with the ARRIVE guidelines.

### 2.2 Blood sampling and muscle tissue extraction

Eight weeks after the start of the experimental procedure (∼24 h after the last running activity in the trained groups), all the mice in the sedentary and trained groups (Figure 1) were decapitated after cervical dislocation. Blood samples taken from the jugular veins were collected in plain tubes containing EDTA and then centrifuged at 653 g for 15 min at 4°C. Blood plasma was stored at −80°C. Immediately after collecting the blood, heart ventricles were dissected and frozen in liquid nitrogen.

### 2.3 Targeted metabolomic analysis and metabolite quantification

For targeted metabolomic analysis and metabolite quantification, plasma and heart samples were prepared and analyzed following the previously used protocol (see Majerczak et al., 2022). The concentrations of branched-chain amino acids ([BCAAs]) including ([valine]) ([leucine]) ([isoleucine]) and the concentration of selected tricarboxylic acid cycle (TCA) intermediates ([TCAi]) including ([citrate]) ([isocitrate]), α-ketoglutarate (2-oxoglutarate) ([α-KG]) ([succinate]) ([fumarate]) and ([malate]) were examined in plasma and in the heart samples by LC–MS/MS-targeted metabolomic method as described previously, with minor changes (Kus et al., 2018). The levels of the studied metabolites measured in the heart tissue samples were normalized to the mg of total protein (Majerczak et al., 2022). The concentration of total proteins in samples was obtained using Pierce™ BCA Protein Assay Kit (Cat# 23225, Thermo Scientific™, Waltham, MA, United States). All samples were prepared and analyzed following the manufacturer’s instructions. All standards including isoleucine (Cat# 58880), leucine (Cat# L8000), valine (Cat# 920), citrate (Cat# C0759), isocitrate (Cat# I1252), α-KG (Cat# 75890), succinate (Cat# S3674), fumarate (Cat# 47910), malate (Cat# 02300), were purchased from Sigma-Aldrich (Merck KGaA, Darmstadt, Germany).

In the present study [BCAAs] (both in the plasma and in the heart ventricles) represents the sum of [valine] [leucine] and [isoleucine]. Similarly, the TCAi pool [TCAi] represents the sum of [citrate] [isocitrate] [α-ketoglutarate] [succinate] [fumarate] and [malate].

### 2.4 Citrate synthase activity microplate assay

The heart samples were diluted (1:20) in muscle homogenization buffer 250 mM sucrose (Cat# 84100), 20 mM Tris (Cat #T1503), 40 mM KCl (Cat# P9333) and 2 mM ethylene glycol-bis(2-aminoethylether)-N,N,N′,N′-tetraacetic acid (Cat# E4378, EGTA) all purchased from Merck KGaA, Darmstadt, Germany). Then, the samples were agitated using a rotator HulaMixer™ Sample Mixer (1 s of 60 RPM orbital motion, 90° reciprocal and 5 s of vibrating motion; Thermo Scientific™, Waltham, MA, United States) for 45 min at 4°C. Next, the homogenates were centrifuged for 10 min at 600 × *g*, after which the supernatant was collected. Protein concentrations were measured using a NanoDrop 2000c (Thermo Fisher Scientific™, Waltham, MA, United States). A spectrophotometric enzyme assay was used to measure citrate synthase (CS) activity with an Infinite 200Pro multiplate reader (Tecan, Männedorf, Switzerland) in a 200 µL reaction mixture containing 1 mM 5.5′-di-thiobis-(2-nitrobenzoic) (Cat# D218200, Merck KGaA, Darmstadt, Germany; DTNB) in 100 mM Tris, pH 8.0, 10 mM acetyl-CoA (Cat# A2181, Merck KGaA, Darmstadt, Germany), 200 mM Tris +0.2% Triton X-100 (Cat# T8787, Merck KGaA, Darmstadt, Germany), and 10 μg of homogenate supernatant protein. The reaction was induced by the addition of 10 mM oxaloacetate (Cat# O4126, Merck KGaA, Darmstadt, Germany) and monitored for 90 s at 412 nm at a set temperature of 37°C. CS activity in the heart (expressed as U ^.^ mg^-1^) is presented as the sum of the data collected from two independent experiments conducted with the same paradigm.

### 2.5 Protein extraction and western immunoblotting analysis

Protein analysis of the heart lysates was performed as described previously (Majerczak et al., 2022). The protein concentrations of the samples were measured using a NanoDrop 2000c (Thermo Scientific™, Waltham, MA, United States). Heart extracts were stored at −80°C until analysis. Equal amounts of total protein were loaded on gels. To eliminate differences between the gels resulting from unequal transfer, an internal standard (IS) (*i.e.,* an additional sample from the same muscle from the WT-Sed group) was applied to each gel. After electrophoresis, the proteins were transferred onto nitrocellulose membranes (GE™ Healthcare, Pittsburgh, PA, United States) in transfer buffer at 4°C and at a constant voltage (35 V). Next, Ponceau S staining (0.1% w/v in 5% acetic acid, Merck KGaA, Darmstadt, Germany) was used to ensure equal loading and transfer of proteins, after which the protein bands were detected. After Ponceau staining, the membranes were scanned and then blocked with 5% dry milk in TBST (tris-buffered saline, 0.1% Tween-20). The membranes were incubated with primary antibodies specific for subunits of the electron transport chain (ETC), *i.e.*, subunit SDHB (Complex II), subunit UQCRC2 (Complex III), subunit MTCO1 (Complex IV) and subunit ATP5A (Complex V) (Cat# ab110413, Abcam, Cambridge, United Kingdom), as well as citrate synthase (Cat# ab129095, Abcam, Cambridge, United Kingdom). After the incubation with primary antibodies, membranes were washed and incubated with secondary antibodies conjugated with horseradish peroxidase. The protein bands were visualized by an enhanced chemiluminescence method, the data were imaged using GeneGnome 5 Syngene (GenSys 1.2.7.0; Syngene BioImaging, Cambridge, United Kingdom), and densitometric quantification was performed using Gene Tools software (Syngene Bio Imaging, Cambridge, United Kingdom). The optical density values obtained for the detected proteins were normalized to the internal standard and to the protein content detected with Ponceau staining at 40 kDa as described previously (Majerczak et al., 2022; Majerczak et al., 2024). The obtained protein densitometric data are presented in arbitrary units (a.u.). We used the sum of the subunits of ETC complexes (ETC, proteins) as a marker of mitochondrial content in the heart (the sum of the subunits of complex II, complex III, complex IV, and complex V) (Majerczak et al., 2022; Majerczak et al., 2024). Representative blots and corresponding Ponceau detection results are shown in the Supplementary Material.

### 2.6 Mitochondrial DNA (mtDNA) copy content

DNA was isolated from the heart samples using QIAamp DNA Mini Kit (Qiagen Inc., Valencia, CA, United States) according to the manufacturer’s protocol, and quantified by spectrophotometry using a NanoDrop ND-1000 Spectrometer (NanoDrop Technologies, Inc., Wilmington, DE, United States). The mtDNA content was measured using qPCR with a CFX96 Real-Time system (Bio-Rad Laboratories, Inc., United States). TaqMan assay (Thermo Fisher Scientific™, Waltham, MA, United States) targeting Atp6 mitochondrial gene was used to quantify the mtDNA. The probe and the primers for qPCR mtDNA analysis were designed using File Builder 3.1, and ordered as a custom TaqMan assay (Thermo Fisher Scientific™, Waltham, MA, United States). The TaqMan probe (assay ID CCCSVZ3, MT-ATP6) was labeled with the FAM fluorescent reporter. The quantity of mtDNA was corrected by simultaneous measurement of the single-copy nuclear Tert (telomerase reverse transcriptase) gene. A custom TaqMan assay was used to quantify the nuclear DNA (nDNA) (Custom TaqMan1 Copy Number Assay, Mm00653609_cn Tert; Thermo Fisher Scientific™, Waltham, MA, United States). The PCR reaction was performed as described before (Kryściak et al., 2018). Calibration curves were used to quantify mtDNA and nDNA copy numbers, which were based on the linear relationship between the crossing point cycle values and the logarithm of the starting copy number.

### 2.7 Statistics

The results of this study are presented as the means and standard deviations (SDs). The obtained data were examined for the presence of outliers with the Grubbs test. Two–way analysis of variance (ANOVA) followed by a Tukey *post hoc* test was used to evaluate the impact of disease (factor: heart failure, HF) and spontaneous wheel running (factor: 8-sWR). Additionally, we analyzed the impact of HF and the 8-sWR on the time of running activity per week during the initial 2 weeks (first-second week of running) and during the main stage of the physical activity period (3^rd^-8^th^ week of running) using two-way ANOVA with repeated measures. Statistical analyses with ANOVA were performed after checking the normality of the distribution and the homogeneity of variance. Some variables in the original data were transformed to a logarithmic scale (valine concentration in the heart and plasma, α-ketoglutarate concentration in the heart) to be able to perform valid analysis of variance. In the case of unequal variances (mean velocity during the initial and main stages of 8-sWR, citrate and valine concentrations in plasma and myocardial succinate content), the data were analyzed using Welch ANOVA followed by Games-Howell *post hoc* test. Due to the nonnormal distribution of some variables (distance during the initial and main stages of the 8-sWR and myocardial isocitrate content), the Kruskal–Wallis test followed by a Dunn’s *post hoc* test was used.

To compare the total running performance in the wheels of trained WT and trained Tgα_q_*44 mice, the Mann‒Whitney U test was performed, and 2-sided exact *P* values are shown. Spearman’s rank correlation coefficient was calculated depending on the normality of the pooled data distributions to assess the relationships between variables.

To estimate the sensitivity of the experiment, some ANOVA power calculations were performed. For the balanced 2 × 2 ANOVA design with a total number of 72 observations and for the standard effect sizes f = 0.1, 0.25, and 0.40, conventionally attributed to small, medium, and large effects, the powers of the corresponding F tests were 0.13, 0.55, and 0.92 for the 2-level effects and for the interaction, respectively. *P* < 0.05 indicated statistical significance, and two-tailed *P* values are presented. Statistical analyses of the data were carried out using Origin2022 v 9.9 software (OriginLab Corporation, Northampton, MA, United States).

## 3 Results

### 3.1 Exercise performance in a murine model of chronic heart failure

Exercise performance in the trained groups of mice (spontaneous wheel running) was registered during 8 weeks. The exercise intensity of the spontaneous running in the wheels was of moderate-intensity since it corresponded to 34% and 23% of the maximal instantaneous running velocity (Supplementary Figures S7) of WT and Tgα_q_*44 mice in wheels, respectively (Figure 1). This would correspond in humans to the moderate exercise intensity (fast walking/slow running/jogging).

**FIGURE 2:**
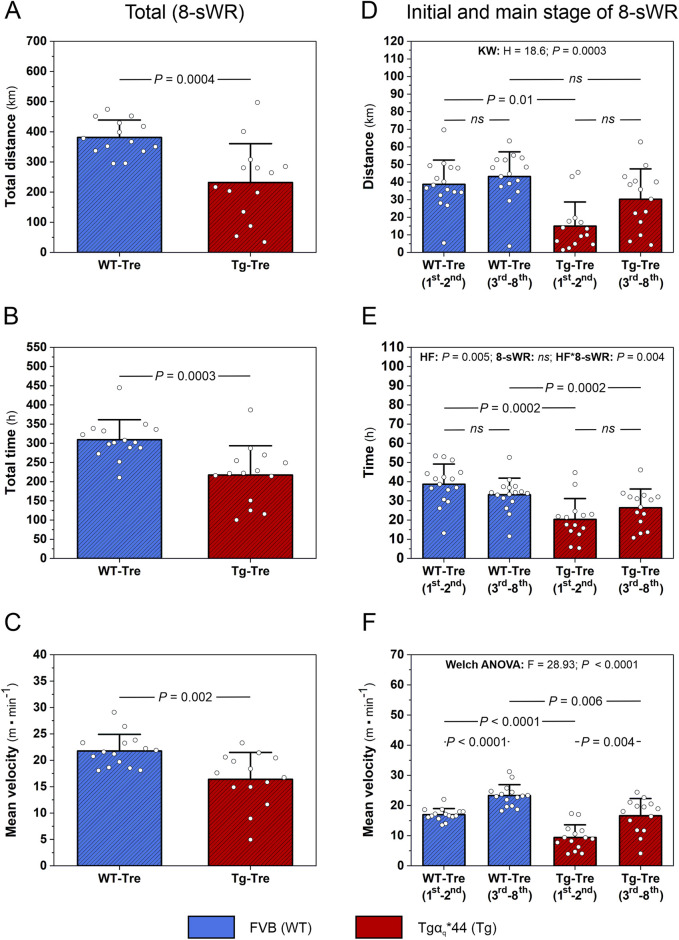
Exercise performance in the wild-type (WT) and in the murine model (Tgα_q_*44) of chronic heart failure (HF) during 8 weeks of spontaneous wheel running (8-sWR). Total distance covered by mice (n = 14–14) **(A)**, total time spent on running (n = 15–14) **(B)** and mean velocity (n = 15–14) **(C)** during 8-sWR. Distance covered per week during the initial stage (first-second week of running) and the main stage (3^rd^-8^th^ week of running) of 8-sWRs (n = 16–15–14–14) **(D)**; time per week spent on running during the initial stage (first-second week of running) and the main stage (3^rd^-8^th^ week of running) of 8-sWRs (n = 16–16–14–14) **(E)**; mean velocity of mice during the initial stage (first-second week of running) and the main stage (3^rd^-8^th^ week of running) of 8-sWRs (n = 15–15–14–14) **(F)**. n indicates the number of analyzed samples for each experimental group in the order: WT-Sedentary (WT-Sed), WT-trained (WT-Tre), Tg-Sedentary (Tg-Sed), Tg-Trained (Tg-Tre). The data are presented as the mean + SD. Each data point in the dot plot represents one individual mouse sample. The Mann-Whitney U test **(A–C)**, Kruskal–Wallis (KW) test followed by a Dunn’s *post hoc* test **(D)**, two-way ANOVA with repeated measures followed by a Tukey *post hoc* test **(E)** and Welch ANOVA test followed by a Games-Howell *post hoc* test **(F)** were used. The statistically significant changes (*P* < 0.05) are plotted on the graphs. *ns*, not statistically significant.

In the present study we found that physical exercise performance of Tgα_q_*44 mice during 8 weeks of spontaneous wheel running (8-sWR) was significantly lower (*P* < 0.05) than that of the WT mice, as reflected by a reduced total distance covered (∼39%, Figure 2A), a shorter total time spent on running (∼30%, Figure 2B) and a lower mean velocity of running (∼25%, Figure 2C).

We also analyzed the distance, time spent on running and mean velocity during the initial 2 weeks (first-second week of running), as well as during the main stage of the physical activity period (3^rd^-8^th^ week of running) (Figures 2D–F). We found that during the initial stage of running, the distance covered by Tgα_q_*44 mice was significantly lower (*P* = 0.01; Figure 2D) than that covered by their control counterparts (similar to the total distance traveled; Figure 2A), whereas during the main stage of running, the distance covered by Tgα_q_*44 mice was not significantly different (*P* > 0.05) from that covered by WT mice (Figure 2D). Furthermore, as reflected by a significant interaction (*P* = 0.004; Figure 2E), the time spent on running by Tgα_q_*44 mice increased (∼29%) from the initial to the main stage of running, whereas in the WT mice, the time spent on running decreased (∼14%) from the initial to the main stage of 8-sWR; however, these differences did not reach statistical significance in *post hoc* analysis (*P* > 0.05; Figure 2E). These results indicate that the physical activity of the Tgα_q_*44 mice in the first 2 weeks of training was markedly lower than that of the WT mice, but in the late stage of training (3^rd^-8^th^ week), the physical activity reached a similar level as that of the WT mice. These findings showed that Tgα_q_*44 mice exhibit adaptive potential for performing physical activity at a comparable level to that of their control counterparts, but they need more time to reach a similar level of physical activity (Figures 2D, E).

### 3.2 The impact of moderate-intensity physical activity on myocardial and systemic BCAAs levels in a murine model of chronic heart failure

**FIGURE 3:**
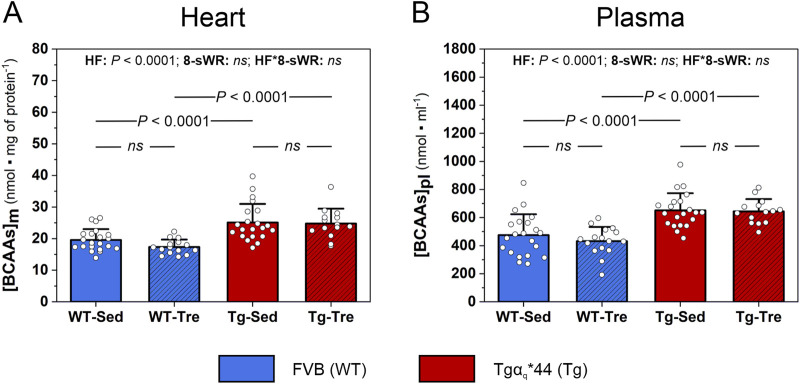
The impact of 8 weeks of spontaneous wheel running (8-sWR) on the myocardial and systemic concentrations of branched-chain amino acids ([BCAAs]) in wild-type (WT) mice and in a murine model (Tgα_q_*44) of chronic heart failure (HF). Myocardial content ([BCAAs]_m_) (n = 21–15–21–14) **(A)**; plasma BCAAs concentration ([BCAAs]_pl_) (n = 21–16–21–14) **(B)**. n indicates the number of analyzed samples for each experimental group in the order: WT-Sedentary (WT-Sed), WT-trained (WT-Tre), Tg-Sedentary (Tg-Sed), Tg-Trained (Tg-Tre). The data are presented as the mean + SD. Each data point in the dot plot represents one individual mouse sample. Two-way ANOVA followed by Tukey *post hoc* test was used. The statistically significant changes (*P* < 0.05) are plotted on the graphs. *ns*, not statistically significant.

The myocardial [BCAAs] in Tgα_q_*44 mice was significantly greater (*P* < 0.05) than that in WT control mice (∼28% and ∼43%, respectively, for the sedentary and trained groups; Figure 3A). Significantly greater [BCAAs] levels were also detected in the plasma of Tgα_q_*44 mice than that of WT control mice (∼37% and ∼49%, respectively, for the sedentary and trained groups; Figure 3B). 8-sWR had no effect on myocardial or systemic [BCAAs] in either the Tgα_q_*44 or WT group (Figures 3A, B). The impact of the disease and 8-sWR on individual myocardial and plasma [BCAAs] is presented in Supplementary Figures S1A–F.

### 3.3 The impact of moderate-intensity physical activity on oxidative metabolism markers in a murine model of chronic heart failure

#### 3.3.1 Myocardial and systemic concentrations of TCA intermediates in a murine model of chronic heart failure

**FIGURE 4:**
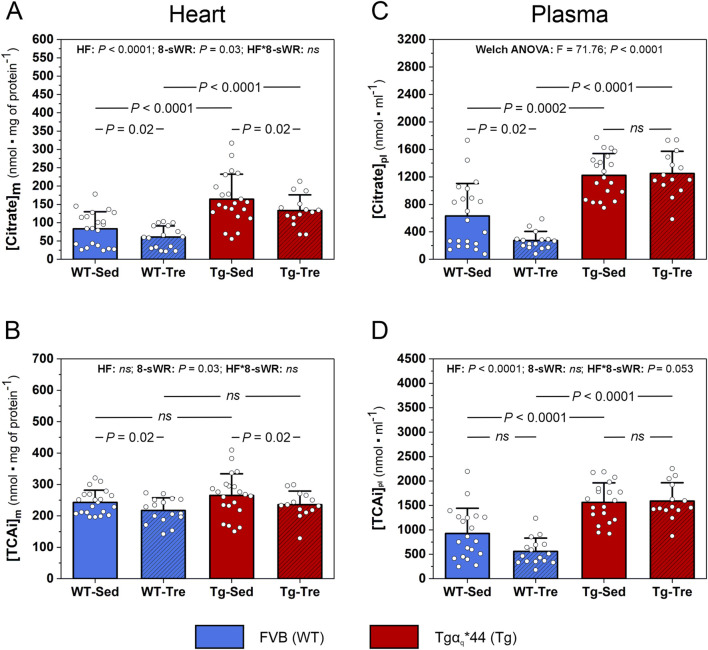
The impact of 8 weeks of spontaneous wheel running (8-sWR) on the myocardial and systemic concentrations of tricarboxylic acid cycle (TCA) intermediates in the wild-type (WT) and in the murine model (Tgα_q_*44) of chronic heart failure (HF). Myocardial citrate content ([Citrate]_m_) (n = 21–16–21–14) **(A)**; the sum of myocardial TCA intermediates ([TCAi]_m_) (n = 21–16–21–14) **(B)**; plasma citrate concentration ([Citrate]pl) (n = 21–15–20–14) **(C)**; and the sum of plasma TCA intermediates ([TCAi]pl) (n = 20–16–19–14) **(D)**. n indicates the number of analyzed samples for each experimental group in the order: WT-Sedentary (WT-Sed), WT-trained (WT-Tre), Tg-Sedentary (Tg-Sed), Tg-Trained (Tg-Tre). The TCAi and citrate concentrations were measured via LC-MS/MS and in case of heart were normalized to total protein content **(A, D)** and presented as absolute concentrations in case of plasma **(C, D)**. TCAi represents the sum of the following intermediates: citrate, isocitrate, α-ketoglutarate, succinate, fumarate and malate concentrations. The data are presented as the mean + SD. Each data point in the dot plot represents one individual mouse sample. Two-way ANOVA followed by a Tukey *post hoc* test **(A–C)** and Welch ANOVA followed by a Games-Howell *post hoc* test **(D)** were used. Statistically significant changes (P < 0.05) were plotted on the graphs.

The percentage distribution (%) of the 6 analyzed TCA intermediates, *i.e.*, citrate, isocitrate, α-KG, succinate, fumarate and malate (TCAi pool), in sedentary WT and Tgα_q_*44 mice is presented in Supplementary Figure S2. In the myocardial TCAi pool, citrate was the most abundant TCA intermediate, constituting ∼34% and ∼62% of the total TCAi pool in the WT and Tgα_q_*44 mice, respectively (Supplementary Figure S2A). As found in the present study myocardial citrate content was significantly greater (*P* < 0.0001) in the Tgα_q_*44 mice than in the WT mice (by ∼97%, Figure 4A). Additionally, the sum of the 6 analyzed TCA intermediates content ([TCAi]) in the heart of the Tgα_q_*44 mice tended to be greater (*P* = 0.07) than that in the heart of the WT control mice (by ∼9%, Figure 4B).

Eight weeks of spontaneous wheel running decreased (*P* = 0.02) the myocardial citrate content to a similar extent (nonsignificant interaction) in both the WT and Tgα_q_*44 mice (by ∼27% and ∼19%, respectively; Figure 4A). Additionally, 8-sWR resulted in a significant decrease in the myocardial TCAi pool (by ∼11%) in both groups (WT and Tgα_q_*44) of mice (*P* = 0.02; Figure 4B). The impact of the disease (HF) and the 8-sWR on the other analyzed myocardial TCA intermediates are presented in Supplementary Figures S3A–E.

Furthermore, we analyzed the concentrations of systemic TCA intermediates and found that, as in the heart, the plasma citrate concentration was also significantly greater (*P* < 0.05) in the Tgα_q_*44 mice than in the WT mice (by ∼94% in the sedentary group and by ∼356% in the trained group; Figure 4C). Similarly, the total TCAi plasma concentration was significantly greater (by ∼69%) in the Tgα_q_*44 mice than in the WT mice (Figure 4D). Interestingly, a significant decrease in the plasma citrate concentration in the WT mice after 8-sWR was observed (by ∼57%, *P* = 0.02; Figure 4C) despite no significant changes in citrate in the Tgα_q_*44 mice (Figure 4C). Furthermore, 8-sWR decreased the plasma [TCAi] in WT mice by ∼40%, whereas in Tgα_q_*44 mice, a slight increase (∼2%) was present (a clear tendency toward a significant interaction; *P* = 0.053, Figure 4D).

#### 3.3.2 Myocardial mitochondrial protein markers in a murine model of chronic heart failure

**FIGURE 5:**
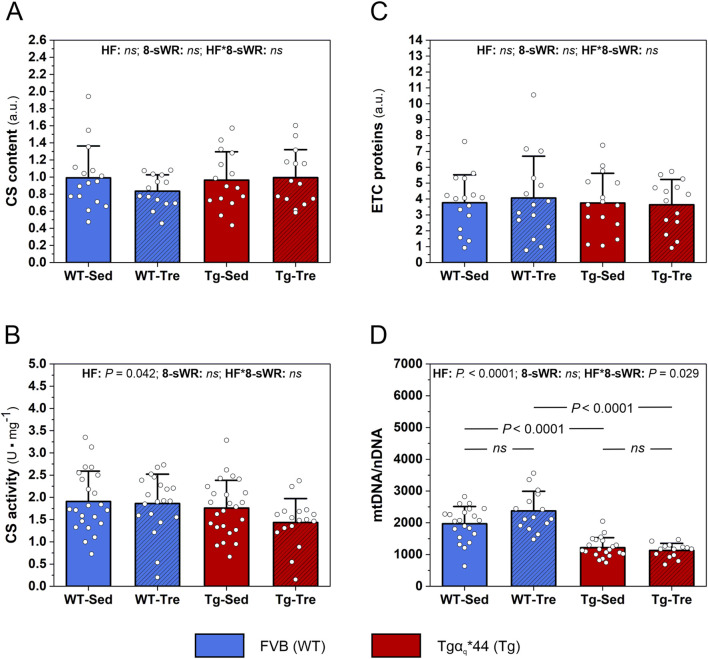
The impact of 8 weeks of spontaneous wheel running (8-sWR) on myocardial oxidative metabolism protein markers in the wild-type (WT) and a murine model (Tgαq*44) of chronic heart failure (HF). Citrate synthase (CS) content (n = 16–14–15–14) **(A)**; CS activity (n = 23–20–25–17) **(B)**; the sum of electron transport chain proteins (ETC.) proteins (n = 16–15–15–14) **(C)** and mtDNA to nDNA ratio (n = 21–15–21–14) **(D)**. n indicates the number of analyzed samples for each experimental group in the order: WT-Sedentary (WT-Sed), WT-trained (WT-Tre), Tg-Sedentary (Tg-Sed), Tg-Trained (Tg-Tre). The data are presented as the mean + SD. Each data point in the dot plot represents one individual mouse sample. Two-way ANOVA followed by a Tukey *post hoc* test was used. Statistically significant changes (*P* < 0.05) were plotted on the graphs. Representative blots and corresponding Ponceau detection results are shown in Supplementary Figures S6A, B.

The myocardial content of the key enzyme of the TCA cycle, citrate synthase (CS), did not differ significantly between the WT and Tgα_q_*44 mice (Figure 5A). However, we observed a clear tendency (*P* = 0.06) toward lower myocardial CS activity in the Tgα_q_*44 mice than in the WT mice (Figure 5B). No impact of 8-sWR on myocardial CS content and activity was found in either of the groups (Figures 5A, B).

We also analyzed the impact of the disease and physical activity on mitochondrial OXPHOS protein markers. We found no significant difference between the WT and Tgα_q_*44 mice in the sum of, ETC proteins (Figure 5C), as well as in the levels of individual, ETC complexes, *i.e.,* complex II, III, IV and V in the heart (Supplementary Figures S4A–D). 8-sWR had no effect on myocardial, ETC proteins content in either the Tgα_q_*44 or WT group (Figure 5C; Supplementary Figures S4A–D).

#### 3.3.3 Myocardial mitochondrial DNA copy number in a murine model of chronic heart failure

We found a significantly lower mtDNA to nDNA ratio (mtDNA/nDNA) in the heart of Tgα_q_*44 mice compared to WT mice, in both sedentary and trained groups (*P* < 0.0001; Figure 5D). Eight weeks of spontaneous wheel running had a different impact on the mtDNA to nDNA ratio in the Tgα_q_*44 mice and WT control group of mice. Specifically, in the control WT mice we observed a nonsignificant increase (by ∼20%) in mtDNA/nDNA ratio, whereas in Tgα_q_*44 mice, 8-sWR was ineffective in changing mtDNA/nDNA ratio (significant interaction, *P* = 0.03; Figure 5D).

### 3.4 Correlations

**FIGURE 6:**
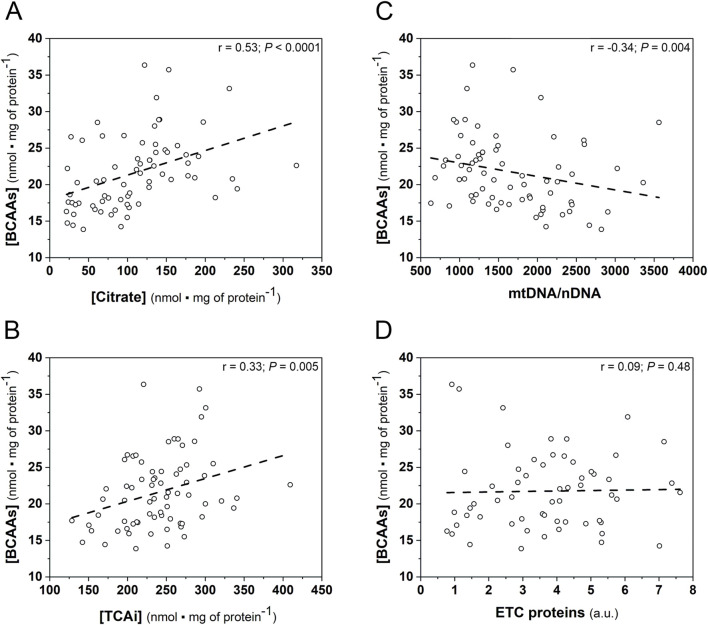
Spearman correlations of the myocardial BCAAs concentration and the oxidative metabolism markers in the hearts of studied mice (WT and Tgα_q_*44 mice) at varied training status. Relationship between BCAAs concentration ([BCAAs]) and citrate concentration ([Citrate]) (n = 71); **(A)**; TCAi concentration ([TCAi]) (n = 71); **(B)**; mtDNA-to-nDNA ratio (n = 71); **(C)** ETC proteins content (n = 59); **(D)**. *Abbreviations:* BCAAs, branched chain amino acids; mtDNA, mitochondrial DNA; nDNA, nuclear DNA; ETC proteins, electron transport chain proteins; TCAi, tricarboxylic acid cycle intermediates.

A significant positive correlation between myocardial BCAAs concentration and the myocardial citrate concentration (Figure 6A), as well as the myocardial sum of TCA intermediates (Figure 6B) was found in the whole group of animals (n = 71).

Moreover, myocardial BCAAs correlated negatively with mtDNA/nDNA ratio (Figure 6C), whereas no relationship between myocardial [BCAAs] ETC., proteins content was observed in the analyzed mice group (Figure 6D).

Additionally, we discovered a significant positive correlation between myocardial BCAAs and systemic BCAAs concentrations (r = 0.44, *P* = 0.0001; Supplementary Figure S5A), as well as a significant positive correlation between myocardial citrate and systemic citrate concentrations (r = 0.60, *P* < 0.0001; n = 71; Supplementary Figure S5B).

In the group of trained animals (n = 29 mice) we found significant negative correlations between total distance covered by mice during the 8-sWR and myocardial, as well as systemic citrate concentration. In addition, a significant positive correlation between total distance and mtDNA copy number was present in the group of trained mice (Table 2).

**TABLE 2:** Correlations of the total distance covered by mice (WT and Tgα_q_*44 mice) during 8 weeks of spontaneous wheel running with the myocardial and systemic oxidative metabolism markers (n = 29).

Total distance (km)		r
Heart	[Citrate]_m_ (nmol ▪ mg of protein^−1^) [TCAi]_m_ (nmol ▪ mg of protein^−1^) CS content (a.u.) mtDNA-to-nDNA ratio ETC proteins (a.u.) [BCAAs]_m_ (nmol ▪ mg of protein^−1^)	−0.49* *ns* *ns* 0,59* *ns* *ns*
Plasma	[Citrate]_pl_ (nmol ▪ ml^-1^) [TCAi]_pl_ (nmol ▪ ml^-1^) [BCAAs]_pl_ (nmol ▪ ml^-1^)	−0.44* −0.45* −0.51*

The symbol * represents significance of the given correlation (*P* < 0.05). *Abbreviations:* [Citrate]_m_ and [Citrate]_pl_, myocardial and plasma citrate concentrations, respectively; [TCAi]_m_ and [TCAi]_pl_, the sum of tricarboxylic acid cycle intermediate concentrations in the heart and in the plasma, respectively; CS, content, citrate synthase content; mtDNA, mitochondrial DNA; nDNA, nuclear DNA; ETC proteins, the sum of electron transport chain proteins; [BCAAs]_m_ and [BCAAs]_pl_, myocardial and plasma branched-chain amino acid concentrations, respectively; *ns*, not statistically significant.

## 4 Discussion

The novel finding of the present study is that myocardial citrate (the most abundant intermediate of Krebs cycle), which is elevated in the failing heart of our murine model of heart failure (Tgα_q_*44 mice), decreased after 8 weeks of moderate-intensity physical activity, reflecting an improvement of the Krebs (TCA) cycle. The current study also demonstrates that the myocardial Krebs cycle is more sensitive to physical activity of moderate-intensity than the mechanism(s) regulating mitochondrial oxidative phosphorylation capacity and BCAAs turnover in the heart.

### 4.1 Myocardial BCAAs in HF

Elevated myocardial and circulating BCAAs levels and their derivatives (*e.g.,* branched-chained ketoacids, BCKAs) have been found in cardiovascular disease, including coronary artery disease (Bhattacharya et al., 2014), myocardial infarction (Wang et al., 2016), dilated cardiomyopathy (Uddin et al., 2019) and heart failure (Hahn et al., 2023). Although the ultimate effect of BCAAs on myocardial function has yet to be determined, nevertheless, there is a growing body of evidence indicating that accumulation of BCAAs in the heart, as a result of impaired oxidative metabolism, may directly reduce the function of heart muscle as a pump (Hahn et al., 2023).

In the present study, consistent with other studies (Hahn et al., 2023), we demonstrated elevated myocardial BCAAs concentration in heart failure, taking advantage of a murine model of chronic heart failure (Tgα_q_*44 mice) (Figure 3A). The elevated BCAAs in the hearts of our Tgα_q_*44 mice, was accompanied by their poorer exercise performance as indicated by (i) a shorter total distance of running during 8 weeks of measurements; (ii) less total time spent on running and (iii) a shorter mean running velocity (Figures 2A–C) compared to WT control mice. The poorer exercise performance of the HF mice was observed both in the initial stage of the observation (first-second week of running) and in the subsequent weeks of physical activity (3^rd^-8^th^ week of running; Figures 2D–F).

The decreased overall exercise performance of the HF mice (Figures 2A–C), which was accompanied by increased myocardial BCAAs levels (Figure 3A), is in agreement with recent findings indicating the role of BCAAs in heart function (Karwi and Lopaschuk, 2023). Accordingly, it was demonstrated that increased myocardial BCAAs levels lower heart contractility through activation of the mTOR-dependent pathway (Portero et al., 2022). This leads to adverse heart remodeling and a decrease in myocardial contractility that may result in a diminution in cardiac output, which is the key factor determining exercise tolerance (Lepretre et al., 2004). Moreover, elevated plasma level of BCAAs (also present in our study, see Figure 3B) has been found to increase susceptibility to arrhythmia (Portero et al., 2022), that also negatively impacts cardiac output and exercise performance.

### 4.2 Myocardial markers of oxidative metabolism in HF

The elevated BCAAs content in the hearts of our HF mice (Figure 3A) can be caused by disturbed oxidative metabolism in mitochondria since BCAAs are ultimately metabolized in the TCA cycle (through the conversion to acetyl-CoA and propionyl-CoA) (Neinast et al., 2019). The TCA cycle in mitochondria is the central metabolic pathway in oxidative metabolism and producer of, *e.g.*, reducing equivalents, which are used along with oxygen to generate ATP in OXPHOS. Therefore, the altered function of the Krebs cycle directly affects energy production in OXPHOS.

In the present study, we found that the content of myocardial citrate which is the most abundant TCA cycle intermediate measured (as confirmed in our study, Supplementary Figures S2A) was significantly greater (by ∼97%) in the Tgα_q_*44 mice than in their control counterparts (Figure 4A). As suggested previously, higher citrate concentration is a sign of the altered Krebs cycle (Williams and O’Neill, 2018), hence, the elevated myocardial citrate content, found in the present study, may confirm an impairment of Krebs cycle in our murine model of HF. Additionally, we found a clear tendency (*P* = 0.06) toward lower CS activity (Figure 4B) in the hearts of Tgα_q_*44 mice than in those of WT control mice in the presence of no difference in myocardial CS content (Figure 5A). That is in agreement with previous data concerning CS content (Bugger et al., 2010) and activity in the failing heart (Gomez-Arroyo et al., 2013). Therefore, our results indicate that in the failing heart an impairment of the Krebs cycle (reflected mainly by increased myocardial citrate levels and decreased CS activity) is accompanied by elevated myocardial BCAAs. Interestingly, Hahn et al. (Hahn et al., 2023), similarly to our study, recently reported higher myocardial BCAAs (especially valine) and citrate concentrations in HF patients with a reduced ejection fraction. Hence, similar to our results, the results of Hahn et al. (2023) showed that higher myocardial BCAAs and citrate levels may indeed be a sign of TCA cycle dysfunction in heart failure patients. Furthermore, we found a greater (by ∼94%, Figure 4C) plasma citrate concentration in the Tgα_q_*44 mice than in the WT control mice. As reported by Stryeck et al. (2019) plasma citrate concentration possesses a prognostic value in heart failure patients, since as demonstrated patients who died within 3 months after the onset of HF had higher serum citrate concentration than patients who survived (Stryeck et al., 2019). It seems to be unlikely that the measured plasma citrate concentration cannot solely represent the myocardial mitochondrial status/TCA cycle activity. Nevertheless, it seems to be more likely that the elevated plasma citrate concentration, as presented in our study, results from an impairment of the TCA cycle activity in the heart itself and in the skeletal muscles (Olkowicz et al., 2021). Indeed, there is a growing body of evidence that the pathological changes in cardiac metabolism is accompanied by an impairment of the skeletal muscle metabolism and its function (Halkar et al., 2020; Olkowicz et al., 2021). Therefore, we consider that the plasma citrate concentration may reflect the impaired TCA cycle activity occurring both in the heart tissue and in the skeletal muscles. A positive correlation between myocardial and plasma citrate concentration, as found in our study (Supplementary Figure S5B), suggests that circulating citrate levels, may have indeed a clinical diagnostic value (as a metabolic marker), as a hallmark of impaired cardiac and skeletal muscle tissue metabolism, especially in HF, as proposed previously (Stryeck et al., 2019).

In the present study, we found no impact of the disease on the mitochondrial OXPHOS protein markers in the heart, such as the, ETC., protein complexes contents (Figure 5C, Supplementary Figures S4A–D). Our results are in contrast to the findings of other studies showing that heart failure is accompanied by changes in the content of myocardial mitochondrial, ETC., protein complexes (Shibayama et al., 2015; Maekawa et al., 2019). However, as suggested, the impact of heart failure on the content of myocardial, ETC., complexes might be related to disease stage (Rosca et al., 2013). Specifically, at the beginning of the illness, *i.e.*, during the compensated hypertrophy period of heart failure, an increase in the content of, ETC., complexes and the OXPHOS rate occur (Rosca et al., 2013), whereas after these initial changes, a progressive decrease in the, ETC., content and OXPHOS activity is observed (Rosca et al., 2013). In the present study, Tgα_q_*44 mice, which are characterized by delayed progression of the disease (Table 1), were examined at the inflection point of the heart failure stage, *i.e.*, in the transition phase from the compensated to decompensated stage of HF, when changes in, ETC., proteins may not be yet visible.

Despite no difference in, ETC., proteins content, we found a significantly lower mitochondrial DNA copy number in our murine HF model compared to control counterparts (∼38%, Figure 5D). Mitochondrial DNA encodes 13 essential subunits of the, ETC., and ATP synthase, making mtDNA indispensable for OXPHOS (Herbers et al., 2019). The increased replication of mtDNA and consequent synthesis of proteins assembled into OXPHOS complexes (Andres et al., 2017; Golpich et al., 2017; Popov, 2020) reflect enhanced mitochondrial biogenesis (Fernández-Vizarra et al., 2011; Golpich et al., 2017). Our results suggest, that the myocardial mitochondrial biogenesis at basal state in our HF mice is in fact impaired and precedes the changes in, ETC., proteins content. Interestingly, a similar decrease in mtDNA copy number (∼40%) has been found in the failing hearts of end-stage HF patients (Karamanlidis et al., 2010). Moreover, based on the data presented earlier by other authors (Fernández-Vizarra et al., 2011), showing that the mtDNA/nDNA ratio positively corresponds with the cytochrome-*c* activity, we cannot exclude the possibility that a decreased myocardial mtDNA/nDNA ratio in our HF mice indicates an attenuated myocardial OXPHOS capacity. Studies of other authors showed that heart failure is indeed accompanied by an attenuation of myocardial OXPHOS activity, reflected by a lower respiration both in myocardium (Maekawa et al., 2019; Takada et al., 2022) and in skeletal muscles (Marın-Garcıa et al., 2001). On the other hand it is suggested that heart seems to have excess of mtDNA copies then are needed for the maintenance of OXPHOS (Pohjoismäki and Goffart, 2017). Nevertheless, a decreased myocardial mtDNA/nDNA ratio in the Tgα_q_*44 mice (Figure 5D) seems to be a feature of the HF-related myocardial mitochondrial dysfunction (Golpich et al., 2017), which may limit exercise tolerance. Therefore, our results support the hypothesis that in heart failure the impaired myocardial oxidative metabolism in mitochondria, reflected by elevated myocardial citrate content (Figure 4A) and by a decreased mtDNA/nDNA (Figure 5D), is discerned to exert an impact on myocardial fuel oxidation (including BCAAs) leading to myocardial BCAAs retention (Figure 3A) and in consequence contributes to the heart dysfunction via BCAAs-dependent mTOR pathway activation (Karwi and Lopaschuk, 2023). Additionally, significantly negative correlation between myocardial mtDNA-to-nDNA and myocardial BCAAs concentration (Figure 6C) suggest that an increase in mitochondrial biogenesis enhances BCAAs oxidation. This result might imply that an increase in mitochondrial biogenesis after physical activity in skeletal muscle (Holloszy, 1967; Hoppeler et al., 1985) and heart (Ventura-Clapier et al., 2007; Vettor et al., 2014), by improving oxidative metabolism, might lead to a decrease in BCAAs concentration and downregulation of the BCAAs-related activation of mTOR pathway. Therefore, in the present study we applied spontaneous wheel running that corresponds to moderate-intensity exercise in humans, such as fast walking, slow running and/or jogging (Figure 1).

### 4.3 The impact of moderate-intensity physical activity on the myocardial oxidative metabolism markers and myocardial BCAAs concentration in HF

Regular physical activity exerts many beneficial effects on cardiovascular health through, *e.g.*, attenuating the main cardiovascular risk factors (hyperlipidemia, obesity, hyperglycemia, hypertension), improving endothelial glycocalyx layer integrity (Majerczak et al., 2017) and increasing nitric oxide bioavailability (for review see Fiuza-Luces et al., 2013). All the afore-mentioned positive effects of physical activity (mainly that of moderate-intensity) may lead to a slowing of heart failure progression and to an improvement of exercise tolerance in HF patients (Sachdev et al., 2023). However, although physical activity delays the progression of HF (Bardi et al., 2019; Wang et al., 2019), the extent of therapeutic effect may depend on the stage of the disease (Bardi et al., 2019).

It is worth adding, that one of the important training-induced adaptations is mitochondrial biogenesis (Holloszy, 1967; Hoppeler et al., 1985) that is reflected by an increase in the mitochondrial biogenesis markers including, ETC., proteins activity/content (Zoladz et al., 2014; 2022; Majerczak et al., 2024), mtDNA copy number (Zoladz et al., 2014; Andres et al., 2017; Popov, 2020) and others (Zoladz et al., 2022). The training-induced enhancement of mitochondrial biogenesis both in skeletal muscles (Holloszy, 1967; Hoppeler et al., 1985) and in the heart (Ventura-Clapier et al., 2007; Vettor et al., 2014), improves energy metabolism (Dudley et al., 1987; Zoladz et al., 2006) and also, in case of HF patients, may lead to an enhancement of exercise tolerance.

In the present study, we found no impact of moderate-intensity physical activity on the, ETC., proteins content (Figure 5C). Furthermore, our results revealed that the 8-sWR was ineffective in increasing mtDNA-to-nDNA ratio in the failing heart of our Tgα_q_*44 mice, whereas at the same time a nonsignificant increase (by ∼20%) in their control counterparts (WT mice) was observed (Figure 5D). This result may suggest that heart failure disrupts mitochondrial biogenesis in the heart, and that this disruption is resilient to the short-term physical activity of moderate-intensity.

Nevertheless, despite no impact of 8-sWR on the OXPHOS markers, we found a decrease in the myocardial TCAi pool after spontaneous wheel running (Figure 4B), including a significant decrease in the citrate concentration (Figure 4A). Therefore, our results show that the Krebs cycle intermediates levels, especially myocardial citrate content, contrary to other markers of oxidative metabolism (*i.e.,* ETC., proteins, mtDNA-to-nDNA ratio) are sensitive even to a moderate-intensity physical activity (Figures 5A–D).

Our results might imply that elevated myocardial TCA cycle intermediates (Figure 4B) including citrate concentration (Figure 4A) in HF is a consequence of a lowered ATP usage due to a disease-related impairment of the heart contractility manifested, e.g., by modification at myofibrillar proteins level in HF (Palmer, 2005). The observed attenuation of the myocardial citrate concentration (Figure 4A) and TCAi pool (Figure 4B) after physical activity in HF mice speaks in favor of this hypothesis. Namely, the training-induced improvement in heart contractility leading to an increase of its mechanical work capacity and enhancement of energy utilization may lead to the increase in myocardial TCA cycle flux intermediates and to an improvement of energy status of the heart. In this way training may improve muscle energy homeostasis in the HF heart in the direction observed in the healthy heart, that possess almost perfect match between ATP supply and ATP usage (Balaban et al., 1986; Korzeniewski et al., 2005).

Additionally, the training-induced decrease in the elevated myocardial citrate content may be an early sign of an improvement of oxidative metabolism in the heart and what is more, a significant positive correlation between myocardial and systemic citrate content found in our study (Supplementary Figure S5B) support the suggestion that systemic citrate concentration may be a useful and sensitive marker of an improvement of oxidative metabolism in the failing heart.

The present study also showed that moderate-intensity physical activity was ineffective in a lowering of the elevated myocardial BCAAs concentration (Figure 3A), however it optimized myocardial mitochondrial Krebs cycle in Tgα_q_*44 mice (Figures 4A, B). It seems that training program of longer duration or performed at higher exercise intensities would be more effective in the lowering of the elevated myocardial BCAAs content in heart failure. Interestingly, as demonstrated by Wu et al. (2022), indeed a more intense training (swimming training, with exercise bouts performed for 90 min, 5 days per week) of the same duration as in our training period (8 weeks) resulted in a decrease of myocardial BCAAs concentration in wild type mice. Moreover, the authors showed that a decrease in myocardial BCAAs content after swimming training was accompanied by an increase (by about 48%) of the myocardial activity of the BCKD - a rate-limiting enzyme of BCAA catabolism. Therefore, those results clearly showed that more intense training program attenuates BCAAs concentration in the heart and this is a consequence of increased myocardial BCAAs oxidation, at least in wild type mice model. Nevertheless, one should take into account that in case of patients (especially patients suffering of cardiovascular diseases) or individuals with lower exercise capacity (as the aged individuals) the exercise of vigorous intensity should be avoided in order to reach health-related benefits of physical activity. It has been demonstrated that high-intesity physical training may disrupt glucose tolerance and insulin secretion even in young healthy individuals (athletes) (Flockhart et al., 2021). Moreover, as recently demonstrated on animal model, physical exercise may lead to excessive cardiac lipid uptake and lipotoxicity, especially when training is performed at high exercise intensities and under high calorie diet (Geng et al., 2025). In consequence, high-intensity training has been found to be associated with a negative training outcomes, such as an enhanced progression of coronary artery calcification and atherosclerotic plaques (Aengevaeren et al., 2023). Therefore, it seems that high-intensity exercise bouts should be avoided in case of patients (especially HF patients) and/or in the group of untrained people (especially in aged individuals).

In the present study, unfortunately we have no performed cardiac function measurements however, as demonstrated in our previous papers (Grassi et al., 2017; Bardi et al., 2019), 8 weeks of spontaneous wheel running in Tgα_q_*44 mice (at similar age as in the present experiment) with a similar workload (total distance covered by Tgα_q_*44 mice ∼206 km) decelerated the progression of the disease. Specifically, Tgα_q_*44 mice developed an improvement of cardiac function (based on cardiac MRI measurements) after 8 weeks of moderate intensity physical activity, i.e., a higher stroke volume (by ∼23%) and ejection fraction (by ∼14%) compared to their untrained counterparts (Table 1) (Bardi et al., 2019). This retardation of HF progression in Tgα_q_*44 mice after moderate-intensity physical activity, may be related to an exercise-induced normalization/optimization of Krebs cycle, as found in the present study in which total distance covered by Tgα_q_*44 mice was even greater (∼232 km, Figure 4B). Summing up, the results of the present study in connection with above-cited study suggest a close association between the training-induced improvement in cardiac tissue metabolism and improvement in heart function.

## 5 Conclusion

In conclusion, in the present study we showed that heart failure is accompanied by an impaired mitochondrial biogenesis, as judged by the lower mtDNA-to-nDNA ratio compared to control mice. Moreover, we have demonstrated for the first time, that physical activity of moderate-intensity, which results in a normalization of elevated myocardial TCA cycle intermediates including lowering of citrate levels, could be considered an important intervention leading to an improvement of myocardial oxidative metabolism in heart failure that precedes the changes in the other myocardial oxidative metabolism markers. Additionally, further studies should pay more attention to plasma citrate as a potential diagnostic variable in the treatment of patients with heart failure.

### 5.1 Limitations of the study

Due to the long duration of development of the end-stage phenotype of heart failure, only female mice were used in the study. Therefore, we cannot be sure whether the findings of this work are also relevant to male Tgα_q_*44 mice. Moreover, due to the limited number of animals and shortage of harvested tissues, we could not conduct others measurements such as, e.g., mitochondrial respiration or/and TCA flux (via isotope-labeled substrates).

## Data Availability

The datasets presented in this study can be found in online repositories. The names of the repository/repositories and accession number(s) can be found below: https://doi.org/10.57903/UJ/I2VK60.

## Abbreviations

α-KG, α-ketoglutarate; BCAAs, branched-chain amino acids; CS, citrate synthase; ETC., electron transport chain; FFAs, fatty acids; HF, heart failure; LC-MS/MS, liquid chromatography coupled with tandem mass spectrometry; mtDNA, mitochondrial DNA; mTOR, mammalian target of rapamycin; nDNA, nuclear DNA; OXPHOS, oxidative phosphorylation; PCr, phosphocreatine; TCA, tricarboxylic acid cycle; Tg-Sed, sedentary Tgα_q_*44 mice; Tg-Tre, trained Tgα_q_*44 mice; *v*_max_, maximal velocity; WT-Sed, sedentary wild type mice; WT-Tre, trained wild type mice; 8-sWR, 8 weeks of spontaneous wheel running.
