# Supplementary material for: Physical activity of moderate-intensity optimizes myocardial citrate cycle in a murine model of heart failure
Source: Front Physiol. 2025 Apr 2;16:1568060. doi: 10.3389/fphys.2025.1568060 (PMC12000009; doi:10.3389/fphys.2025.1568060)
Supplement: Supplementary file 1 [file DataSheet1.pdf]

Supplementary Material

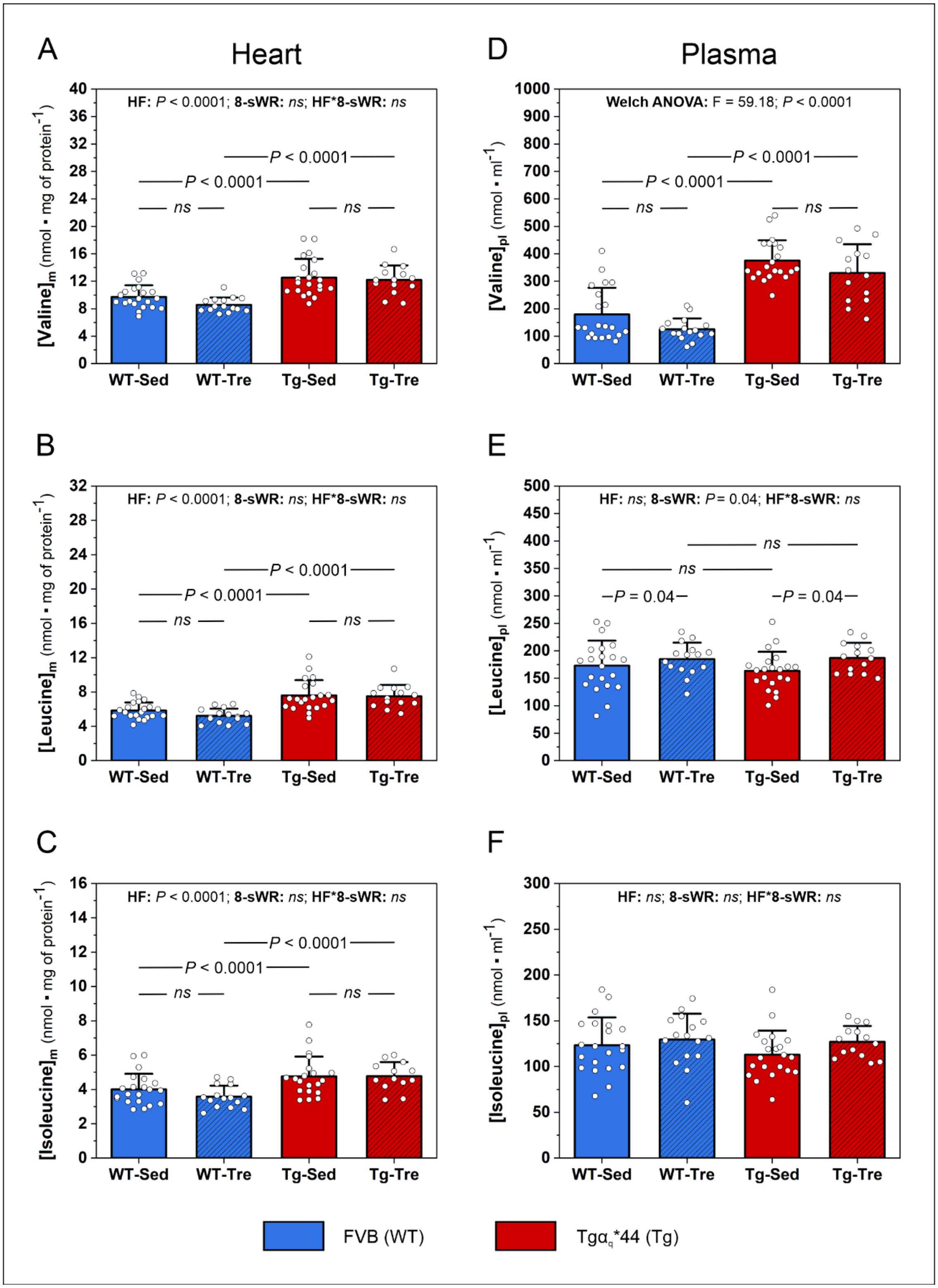

FIGURE S1. The impact of 8-weeks of spontaneous wheel running (8-sWR) on the myocardial and systemic valine, leucine and isoleucine concentrations in the wild-type (WT) and in the murine model (Tgα_q*44) of chronic heart failure (HF). Myocardial valine content ([Valine]$_m$) (n = 21–15–21–14) (**A**); myocardial leucine content ([Leucine]$_m$) (n = 21–15–21–14) (**B**); myocardial isoleucine content ([Isoleucine]$_m$) (n = 21–15–20–13) **(C)**; plasma valine concentration ([Valine]$_{pl}$) (n = 21–16–21–14) (**D**); plasma leucine concentration ([Leucine]$_{pl}$) (n = 21–15–21–14) (**E**); plasma isoleucine concentration ([Isoleucine]$_{pl}$) (n = 21–16–21–14) (**F**). n indicates the number of analyzed samples for each experimental group in the order: WT-Sedentary (WT-Sed), WT-trained (WT-Tre), Tg-Sedentary (Tg-Sed), Tg-Trained (Tg-Tre). Data are presented as mean + SD. Each data point in the dot plot represents one individual mouse sample. Two–way ANOVA followed by a Tukey *post-hoc* test (**A**–**C**, **E**–**F**) and Welch ANOVA test followed by a Games-Howell *post-hoc* test (**D**) were used. Statistically significant changes ($P < 0.05$) were plotted on the graphs. *ns*, not statistically significant.

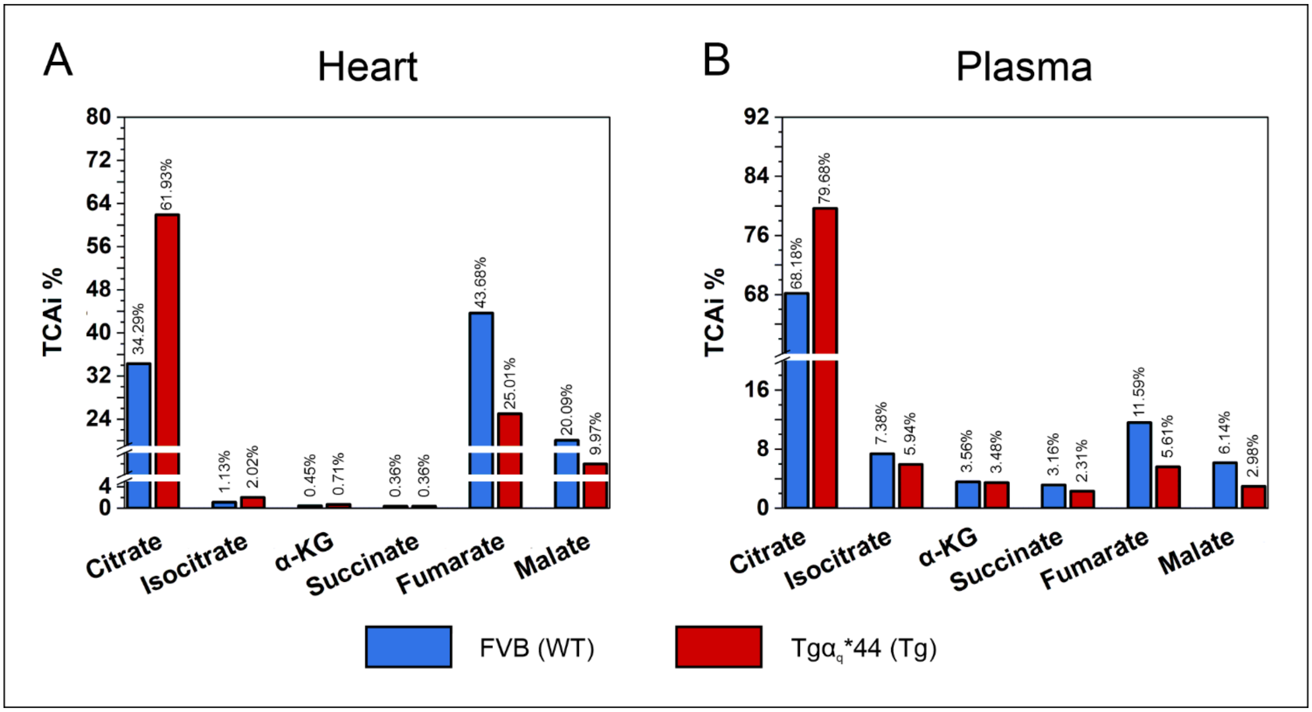

FIGURE S2. Tricarboxylic acid cycle intermediates (TCAi) percentage distribution in the heart (A) and plasma (B) of the sedentary wild-type (WT-Sed) and sedentary Tgα_q*44 (Tg-Sed) mice. ***Abbreviations:*** α-KG, α-ketoglutarate

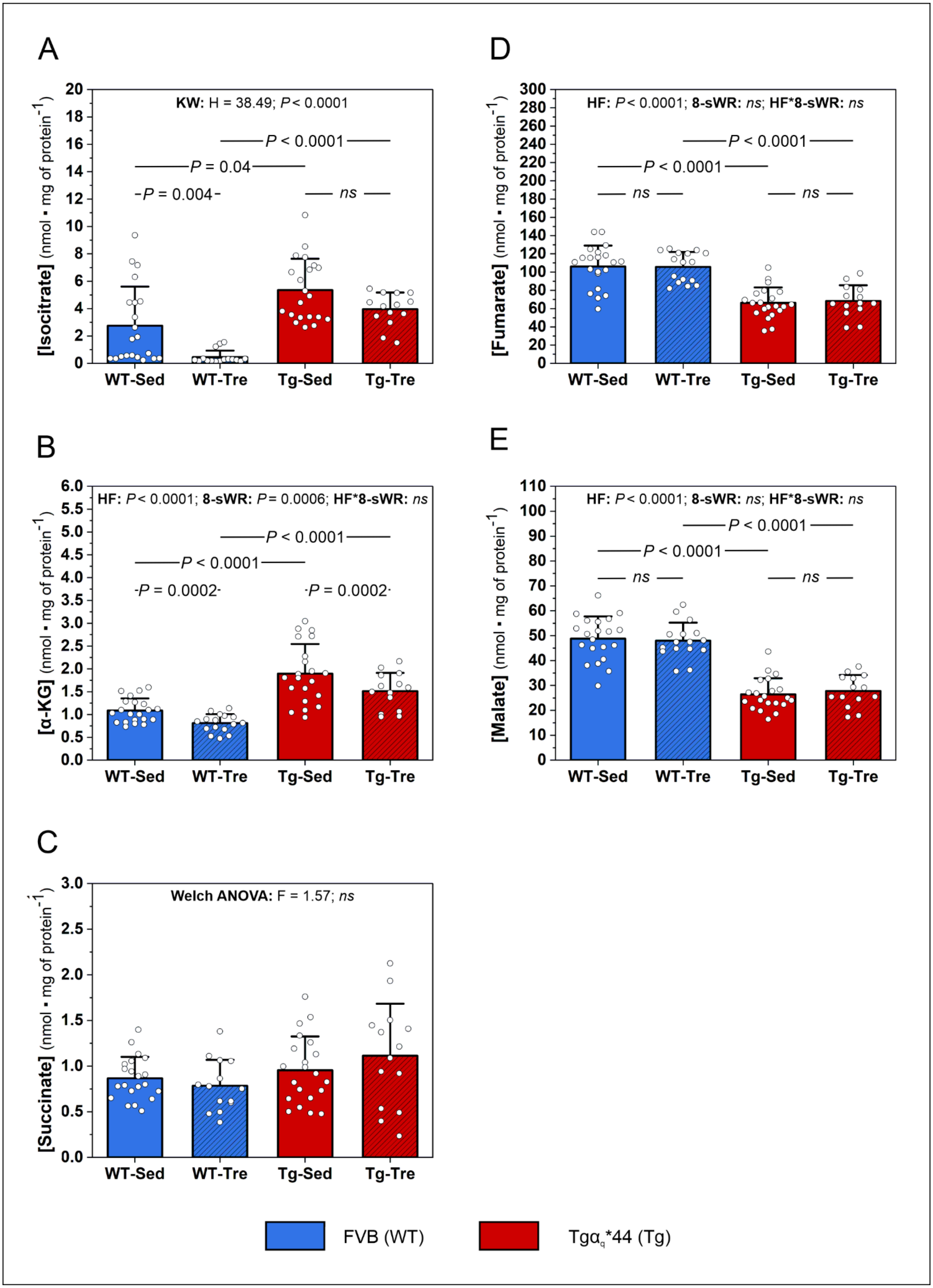
A
KW: H = 38.49; P < 0.0001
P < 0.0001
P = 0.04
P = 0.004
ns
[Isocitrate] (nmol • mg of protein⁻¹)
WT-Sed
WT-Tre
Tg-Sed
Tg-Tre
B
HF: P < 0.0001; 8-sWR: P = 0.0006; HF*8-sWR: ns
P < 0.0001
P < 0.0001
P = 0.0002
P = 0.0002
[α-KG] (nmol • mg of protein⁻¹)
C
Welch ANOVA: F = 1.57; ns
[Succinate] (nmol • mg of protein⁻¹)
D
HF: P < 0.0001; 8-sWR: ns; HF*8-sWR: ns
P < 0.0001
P < 0.0001
ns
ns
[Fumarate] (nmol • mg of protein⁻¹)
E
HF: P < 0.0001; 8-sWR: ns; HF*8-sWR: ns
P < 0.0001
P < 0.0001
ns
ns
[Malate] (nmol • mg of protein⁻¹)
FVB (WT)
Tgαq*44 (Tg)

FIGURE S3. The impact of 8-weeks of spontaneous wheel running (8-sWR) on the myocardial contents of tricarboxylic acid cycle (TCA) intermediates ([TCAi]) in the wild-type (WT) and in the murine model (Tgα$_q$*44) of chronic heart failure (HF). Isocitrate content ([Isocitrate]) (n = 21–16–21–14) (**A**); α-ketoglutarate ([α-KG]) (n = 21–16–21–14) (**B**); succinate content ([Succinate]) (n = 21–14–21–14) (**C**); fumarate content ([Fumarate]) (n = 21–16–21–14) (**D**); malate concentration ([Malate]) (n = 21–16–21–14) (**E**). n indicates the number of analyzed samples for each experimental group in the order: WT-Sedentary (WT-Sed), WT-trained (WT-Tre), Tg-Sedentary (Tg-Sed), Tg-Trained (Tg-Tre). Data are presented as mean + SD. Each data point in the dot plot represents one individual mouse sample. Kruskal Wallis test followed by a Dunn's *post-hoc* test (**A**), two-way ANOVA followed by a Tukey *post-hoc* test (**B**, **D**–**F**) and Welch ANOVA test followed by a Games-Howell *post-hoc* test (**C**) were used. Statistically significant changes ($P < 0.05$) were plotted on graphs. *ns*, not statistically significant.

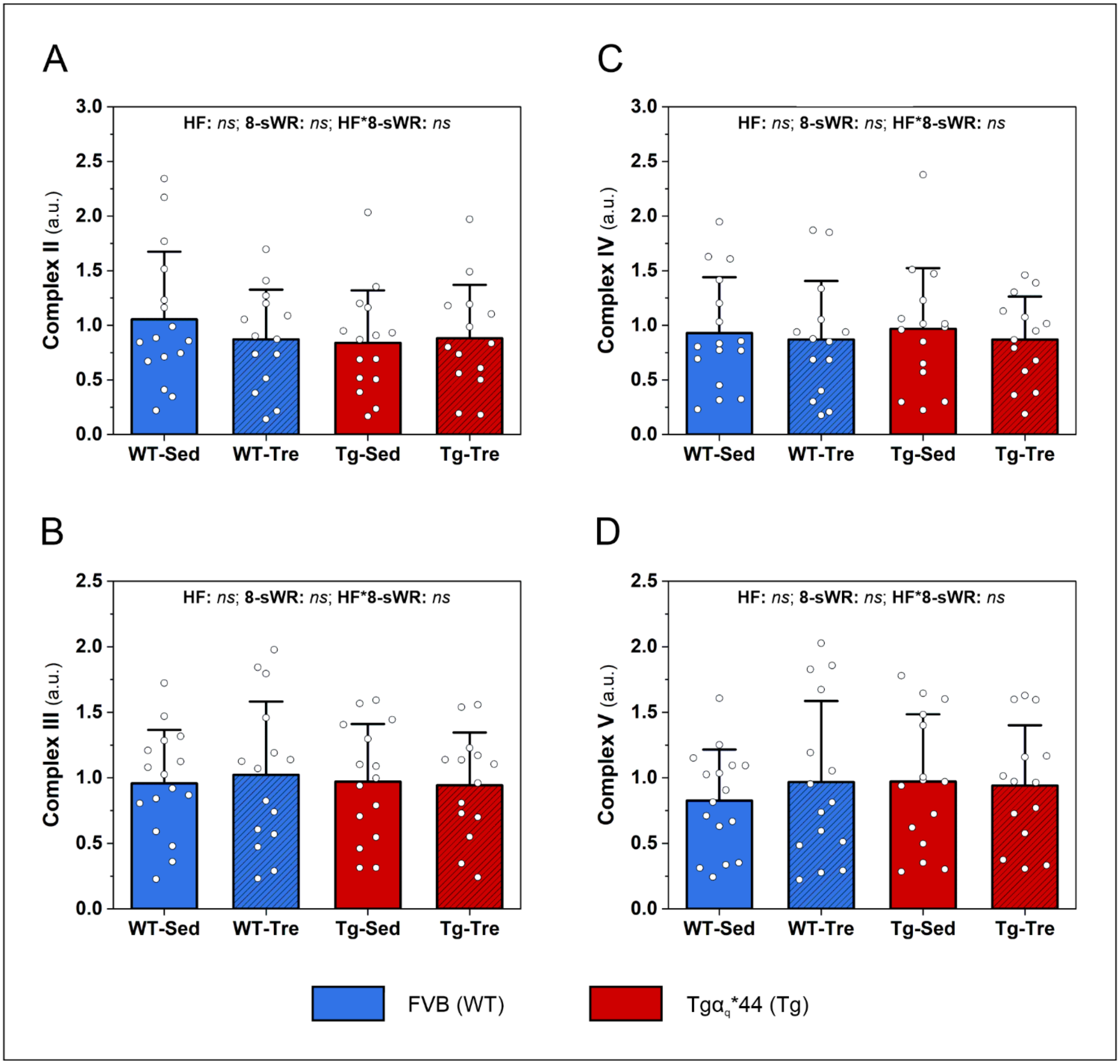

FIGURE S4. The impact of 8-weeks of spontaneous wheel running (8-sWR) on the myocardial electron transport chain (ETC) proteins in the wild-type (WT) and in the murine model (Tgα_q*44) of chronic heart failure (HF). The abundance of: subunit SDHB of ETC complex II (Complex II) (n = 16–14–15–14) (**A**); the subunit UQCRC2 of complex III (Complex III) (n = 16–15–15–14) (**B**); the subunit MTCO1 of complex IV (Complex IV) (n = 16–14–15–14 (**C**); the subunit ATP5A of complex V (ATP synthase, Complex V) (n = 16–15–15–14) (**D**). n indicates the number of analyzed samples for each experimental group in the order: WT-Sedentary (WT-Sed), WT-trained (WT-Tre), Tg-Sedentary (Tg-Sed), Tg-Trained (Tg-Tre). Data are presented as mean + SD. Each data point in the dot plot represents one individual mouse sample. Two–way ANOVA followed by a Tukey *post-hoc* was used. Statistically significant changes ($P < 0.05$) were plotted on the graphs. *ns*, not statistically significant. The representative blots and corresponding Ponceau detection are shown in Supplementary **Figure S6.**

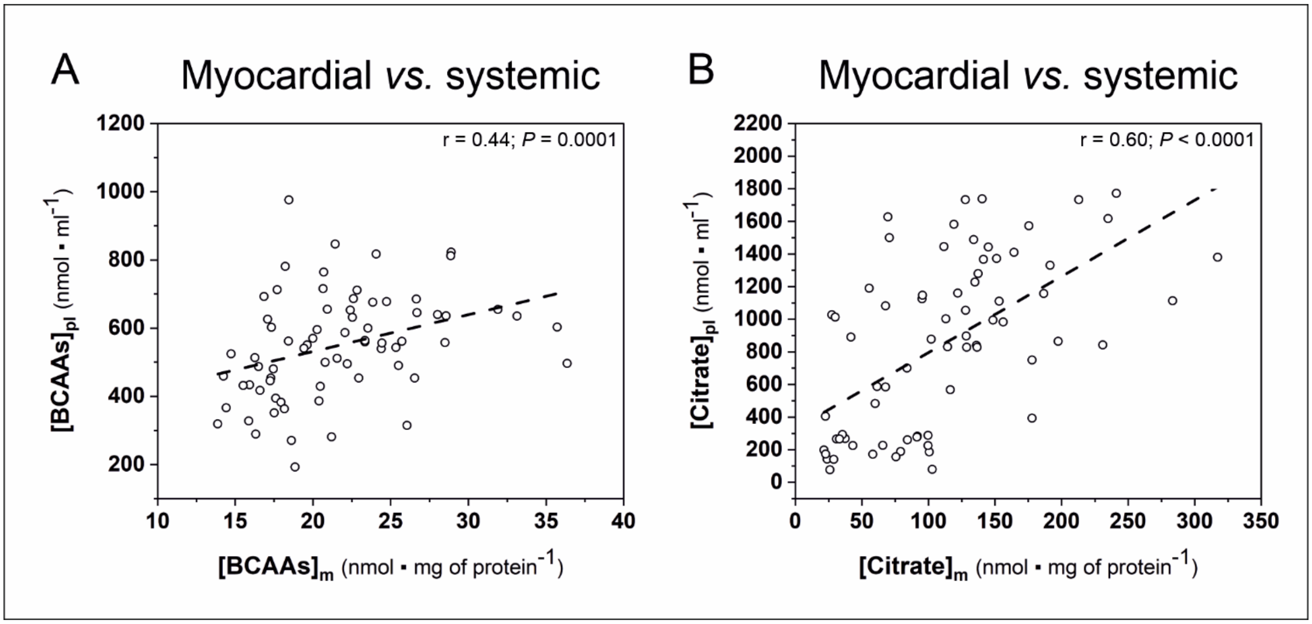

FIGURE S5. Spearman correlations of BCAAs and citrate contents (myocardial *vs* systemic) of the studied mice (WT and Tgα_q*44 mice) at varied training status. Relationship between myocardial and systemic BCAAs concentrations ([BCAAs]) (n = 71) (**A**) and between myocardial and systemic citrate concentrations ([Citrate]) (n = 71) (**B**). ***Abbreviations:*** $[BCAAs]_m$ and $[BCAAs]_{pl}$, myocardial and plasma [BCAAs], respectively; $[Citrate]_m$ and $[Citrate]_{pl}$, myocardial and plasma [Citrate], respectively.

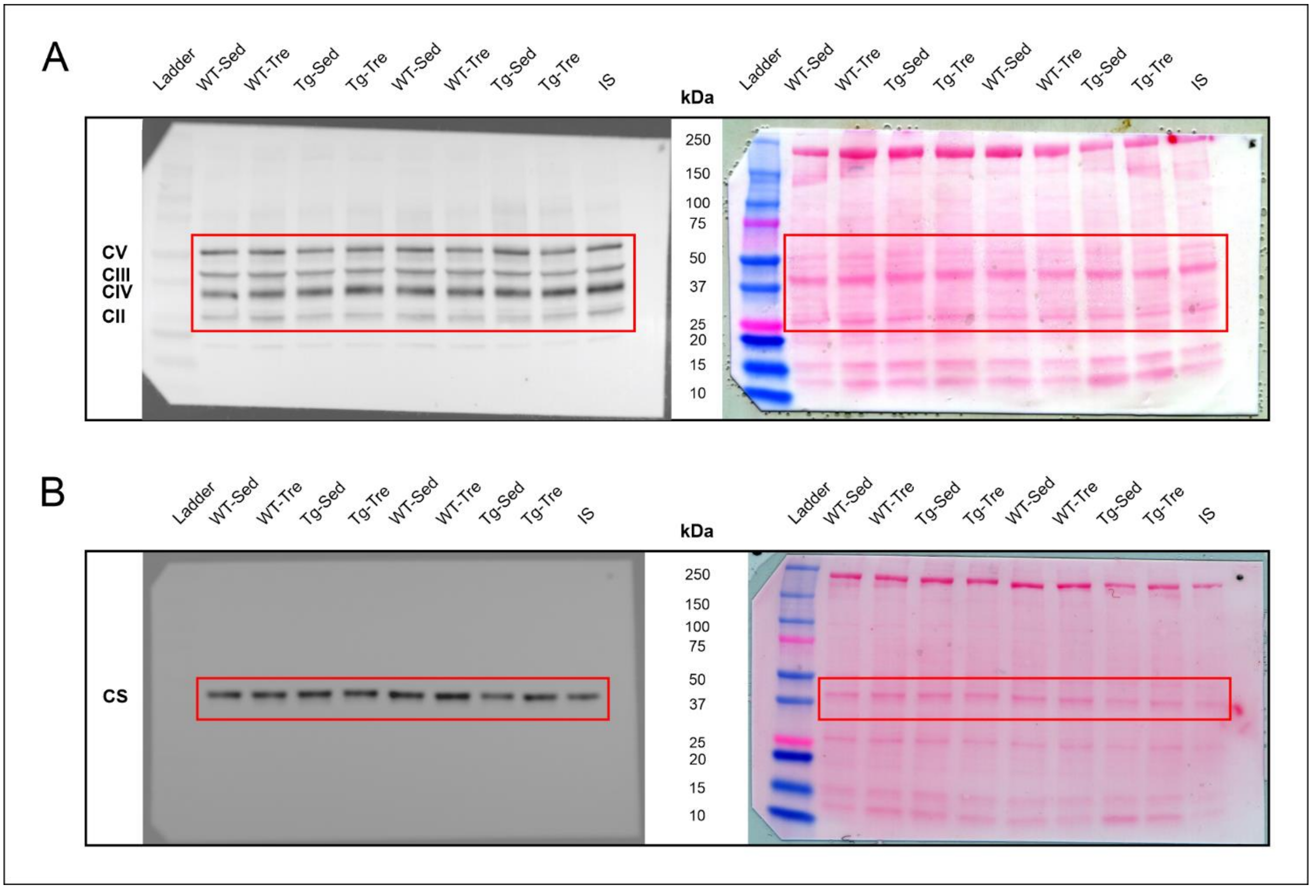

FIGURE S6. The representative immunoblot demonstrating detection of subunits of electron transport chain complexes (A) and citrate synthase (CS) (B) in the heart of the wild-type (WT) and of the murine model (Tgα_q*44) of chronic heart failure. Ponceau S staining (right side) of the same membranes demonstrating total protein loaded. The protein ladder is a visible Precision Plus Protein Dual Colour Standards (Bio-Rad, Cat#1610374). The internal standard (IS) is a mice heart sample of WT-Sed group.

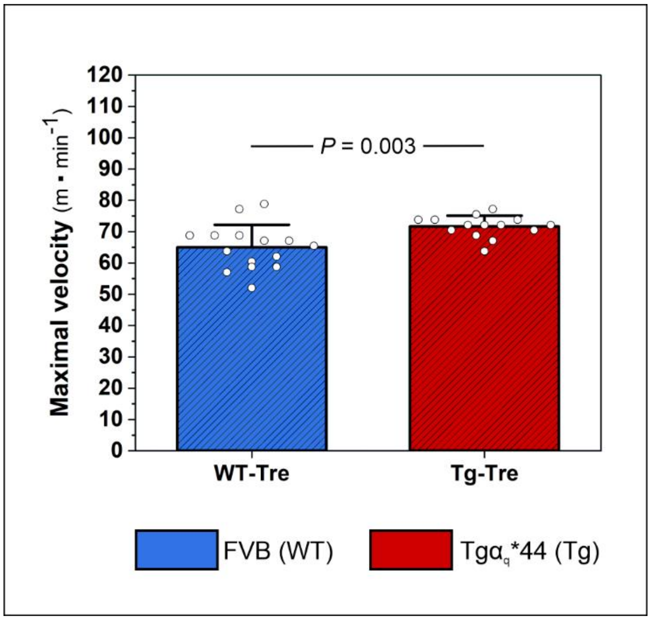

FIGURE S7. Maximal velocity of running during 8 weeks of spontaneous wheel running (8-sWR) performed by the wild-type (WT) and the murine model of chronic heart failure (HF) (Tgα_q*44) mice. (n = 15–14), n indicates the number of analyzed samples for each experimental group in the order: WT-trained (WT-Tre), Tg-Trained (Tg-Tre). The data are presented as the mean + SD. Each data point in the dot plot represents one individual mouse sample. The Mann-Whitney U was used. The statistically significant change ($P < 0.05$) is plotted on the graph.
